# IL-26, a Cytokine With Roles in Extracellular DNA-Induced Inflammation and Microbial Defense

**DOI:** 10.3389/fimmu.2019.00204

**Published:** 2019-02-12

**Authors:** Vincent Larochette, Charline Miot, Caroline Poli, Elodie Beaumont, Philippe Roingeard, Helmut Fickenscher, Pascale Jeannin, Yves Delneste

**Affiliations:** ^1^CRCINA, INSERM, Université de Nantes, Université d'Angers, Angers, France; ^2^CHU Angers, Département d'Immunologie et Allergologie, Angers, France; ^3^Inserm unit 1259, Medical School of the University of Tours, Tours, France; ^4^Institute for Infection Medicine, Christian-Albrecht University of Kiel and University Medical Center Schleswig-Holstein, Kiel, Germany

**Keywords:** IL-26, inflammation, kinocidin, soluble PRM, DNA carrier

## Abstract

Interleukin 26 (IL-26) is the most recently identified member of the IL-20 cytokine subfamily, and is a novel mediator of inflammation overexpressed in activated or transformed T cells. Novel properties have recently been assigned to IL-26, owing to its non-conventional cationic, and amphipathic features. IL-26 binds to DNA released from damaged cells and, as a carrier molecule for extracellular DNA, links DNA to inflammation. This observation suggests that IL-26 may act both as a driver and an effector of inflammation, leading to the establishment of a deleterious amplification loop and, ultimately, sustained inflammation. Thus, IL-26 emerges as an important mediator in local immunity/inflammation. The dysregulated expression and extracellular DNA carrier capacity of IL-26 may have profound consequences for the chronicity of inflammation. IL-26 also exhibits direct antimicrobial properties. This review summarizes recent advances on the biology of IL-26 and discusses its roles as a novel kinocidin.

Cytokines are a class of signaling molecules expressed by many cell types, and especially those of the immune system. They are classified in seven major families: the type I cytokine, the type II cytokine (1), the IL-1 (2), and the TGF-β (3) families, the TNF superfamily (4), the receptor tyrosine kinase cytokine family (5), and the chemokine family (6). Although exhibiting pleiotropic properties, they are pivotal regulators of innate and adaptive immune defenses, inflammation, and hematopoiesis.

The term “interleukin” (IL) originally refers to a group of cytokines expressed by leukocytes. To date, interleukins have been reported expressed by a wide variety of immune and non-immune cells and exhibit a large panel of properties (e.g., affecting proliferation, activation, differentiation, maturation, migration, and adhesion). Although agonist/antagonist activities and redundancy make any classification particularly complicated, different classifications have been proposed based on their functions, receptor usage or structure. As an example, a functional classification distinguishes eight subgroups of interleukins (IL-1, common γ chain receptor cytokine, cytokines of type 2 immune responses, interleukins with chemokine activity, the IL-10, IL-12, and IL-17 families and, others) (7). A structure-based classification has been also proposed, dividing interleukins into four major groups: IL-1-like cytokines, class I helical cytokines (IL-4-, IL-6-, and IL-12-like ILs, common γ chain receptor cytokines), class II helical cytokines (IL-10- and IL-28-like molecules) and the IL17-like cytokines (8).

The interleukin 10 (IL-10) cytokine family consists of nine members: IL-10, IL-19, IL-20, IL-22, IL-24, IL-26, and the more distantly related IL-28A, IL-28B, and IL-29 (also named type III interferons) (9). Except IL-10 which exhibits immunoregulatory properties, most members of the IL-10 cytokine family are involved in mucosal immunity (i.e., local immunity, maintenance/restoration of tissue homeostasis, tissue repair), such as IL-22 involved in gut homeostasis, and local immunity (10).

Interleukin 26 (IL-26) is one the most recently identified member of the IL-10 cytokine family (11). Initial studies reported an elevated expression in human chronic inflammatory diseases, leading to a proposal that it may constitute a novel inflammatory mediator (12, 13). Although the absence of IL-26 in rat and mouse has strongly hampered our understanding of its biology, recent reports have shed new light on the functions of IL-26, revealing that it exhibits conventional, and non-conventional cytokine properties. This review summarizes recent data on the biology of IL-26 and its potential pathological roles in inflammatory disorders.

## The IL-26 Protein and Signaling Elements

### Gene Organization

Firstly named AK155, IL-26 was identified as a transcript over-expressed in *Herpesvirus saimiri* (*HVS*)-infected human T-cells (11). Due to sequence (≈25% homology and 47% similarity) and predicted secondary structure similarities with IL-10, AK155 was categorized in the IL-10 cytokine family, a subgroup of the class II helical cytokine family (14).

The organization of the *IL26* gene has been previously detailed (12). Briefly, it is located on chromosome 12 (12q15) (15, 16), between the genes encoding IL-22 and interferon gamma (IFNγ), and contains five exons. *IL26* and *IFNG* genes have the same orientation and share a common enhancer sequence located between the two genes. The *IL26* gene is conserved in various vertebrates (ranging from fish to great apes) and human IL-26 orthologs have been identified in 137 organisms, with the notable exception of mouse and rat (17).

### Protein Characteristics

**Figure 1:**
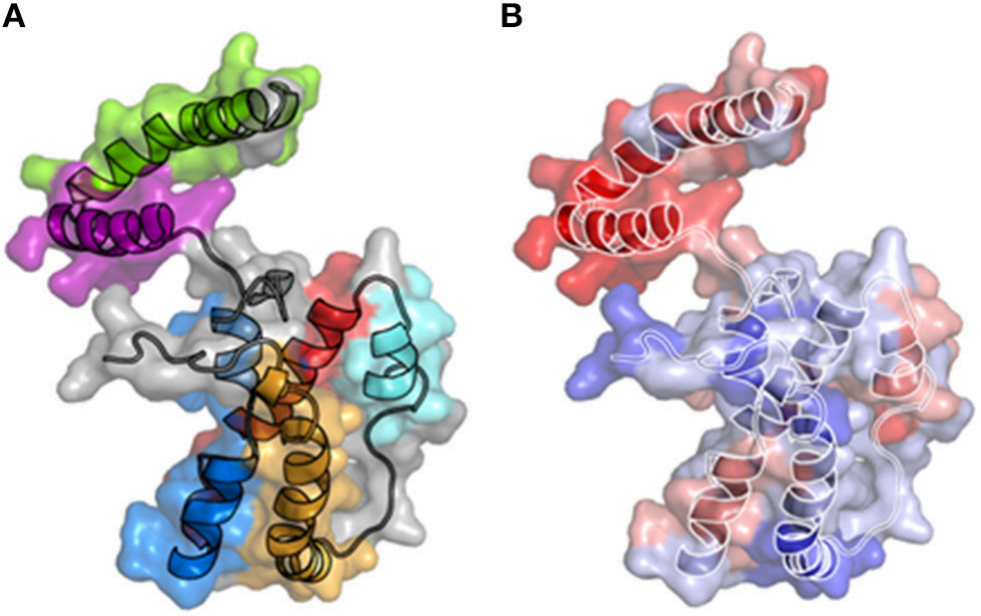
3-D structural models of IL-26. **(A)** A three dimensional structure of IL-26 has been modeled using the IL-10 crystal structure as a template. The six alpha-helices are colored. **(B)** Electrostatic potential on the molecular surface of IL-26 is colored from red (+210 kbT/ec) to blue (+10 kbT/ec). The alpha-helices are shown.

IL-26 exhibits characteristics of cytokines, i.e., six alpha helices (A–F) connected by loops and four conserved cysteines (Figure 1A). The protein comprises 171-amino acids, with a calculated molecular mass of 19,843 Da. Western blotting revealed that recombinant IL-26 has an apparent molecular mass of 19 kDa and that endogenous IL-26 (present in T cell culture supernatants and in human serum) is expressed as a 36 kDa homodimer (11). Meller et al. also predicted a multimeric form of IL-26 (18), but this remains to be confirmed *in vivo*.

IL-26 contains 30 positively charged amino acids (lysine or arginine), conferring to IL-26 unusual physicochemical properties, including a high isoelectric point of 10.77 (11). 3-D modeling reveals the distribution of positively charged residues on the surface of the molecule, providing to IL-26 particular features: the electrostatic potential on the molecular surface is globally positive (11, 19) and, more importantly, the helices E, and F exhibit amphipathic properties (18, 19), with a hydrophic face and a hydrophilic face comprised of positively charged residues (Figure 1B). Consequently, helices E and F, and part of helix C, are enriched in positively charged residues, conferring to IL-26 DNA-binding properties (18, 19). Interestingly, amphipathic α helices containing DNA-binding domains are hallmarks of cationic cell-penetrating peptides (CPP), a family of molecules shuttling extracellular molecules, and especially nucleic acid, into intracellular compartment (20, 21). In agreement, helix F has similarities with the KALA peptide, a synthetic CPP designed for intracellular DNA delivery (20). Consequences of these physicochemical features were only recently understood (18, 19).

### IL-26-Signaling Elements

#### The Conventional IL-26 Receptor

Biological activities of the IL-10 family cytokines are mediated by specific membrane receptors belonging to the cytokine receptor family 2 (CRF2) and comprised of heterodimeric combinations of receptors 1 (IL-10R1, IL-20R1, or IL-22R1) and receptors 2 (IL-10R2 or IL-20R2) (22).

Two independent laboratories identified a conventional receptor for IL-26 (IL-26R), consisting in IL-10R2 and IL-20R1 chains (23, 24). The IL-26-binding site is located within the IL-20R1 subunit and both chains are required for signaling (12). IL-26 triggers Janus kinase 1 (Jak1) and tyrosine kinase 2 (Tyk2) signaling, resulting in the phosphorylation of signal transducer and activator of transcription 1 (STAT1) and STAT3 (23, 24). Interestingly, although IL-10R2 is ubiquitously expressed, IL-20R1 is restricted to epithelial cells (22); IL-20R1 has been also reported expressed by some myeloid cells (25, 26).

#### Other IL-26-Signaling Molecules

Different studies have reported that IL-26 activates innate (27) and adaptive lymphoid cells (28) as well as myeloid cells (29) and plasmacytoid dendritic cells (pDC) (18). However, with the exception of alveolar macrophages (25) and non-stimulated neutrophils (26, 30), immune cells do not express IL-20R1, suggesting the existence of alternative IL-26-signaling pathway(s) in the activation of IL-20R1-negative cells.

A major breakthrough in the biology of IL-26 came from the demonstration that it acts as a shuttling molecule allowing DNA to be internalized by pDC, leading to their activation via the intracellular DNA sensor toll-like receptor 9 (TLR9) (18). We confirmed this unique property by showing that IL-26/DNA complexes activate human monocytes via the inflammasome and the cytosolic stimulator of interferon genes (STING) pathways (19).

Different hypotheses have been proposed to explain how IL-26 may enter into the cytoplasm in the absence of a conventional membrane receptor and there activate innate immune cells. First, IL-26 can bind to glycosaminoglycans (heparin and heparan sulfate) on the plasma membrane (11, 18, 31). Second, helix F was predicted to have an in-plane membrane (IPM) motif anchor (19), a domain involved in the binding of proteins to cell membranes and their subsequent internalization (32). Third, IL-26 exhibits features of cell-penetrating peptides (CPP) that have the ability to translocate through membranes in a non-specific manner (33).

The existence of different signaling pathways is suggested by the fact that monomeric and dimeric IL-26 differ in their abilities to stimulate IL-26R-expressing epithelial cells vs. IL-20R1-deficient immune cells (29). Nevertheless, whether IL-26 may activate cells via the conventional IL-26R and intracellular nucleic acids sensors remains undetermined, especially as these pathways induce similar activation profiles.

### Expression of IL-26

The production of IL-26 by activated primary and transformed T cells and by inflammatory immune and non-immune cells is well-established. However, as reported in recent reviews (13, 34), further sources of IL-26 as well as further IL-26-dependent signaling pathways and phenotypes are to be expected due to the non-conventional properties exhibited by IL-26 (accumulation within cells as a result of its CPP and carrier properties).

#### Expression by Innate and Adaptive Lymphoid Cells

Since the initial discovery of IL-26 expression in human HVS-transformed T cells (11), several studies have confirmed that lymphoid cells, and especially activated T helper 1 (Th1) and Th17 memory CD4^+^ cells, constitute the major source of IL-26. More precisely, IL-26 is expressed by infiltrating pro-inflammatory IL-17-producing T cells present in chronically inflamed tissues from patients suffering from intestinal bowel diseases (IBD) (35) or from joint (29), skin (36, 37), liver (27), or lung inflammation (25, 38, 39). Activated memory CD146^+^ CD45RO^+^ CD4^+^ T cells strongly express IL-26 mRNA compared to CD146^+^ CD45RO^−^ CD4^+^ T cells (40). Moreover, Ohnuma et al. have reported that CD26^+^ CD4^+^ T cells are a major source of IL-26 in a model of graft-vs.-host disease (GVHD) (39). IL-26 has also been reported expressed by CD8^+^ T cells (25, 41). In contrast, CD4^+^ T cells polarized toward a regulatory phenotype (Treg) and Th2 lymphocytes have low or no expression of IL-26 (42). Finally, naive CD4^+^ T cells also express IL-26, but at a lower level compared to memory CD4^+^ T cells (42). Thus, IL-26 has emerged as a marker of highly differentiated Th17 cells (36).

The nature of the signals required for IL-26 expression by human T cells remains largely unknown. IL-1β and IL-23, two cytokines crucial for the generation of human Th17 cells (43), induce IL-26 production by CD4^+^ T lymphocytes (44, 45) and, conversely, IL-26-stimulated CD4^+^ T cells secrete IL-17, and IL-23 (46). This loop of cytokine induction may participate in the maintenance of the pro-inflammatory phenotype of infiltrating memory T cells within IL-26-containing inflamed tissues. Interestingly, the expression of IL-26 by CD4^+^ T cells can be induced via signaling pathways other than CD3 and CD28 [(42) and personal unpublished data]. Accordingly, cord blood human CD4^+^ T cells acquire IL-26 expression upon interacting with CD26 with its ligand caveolin-1 (39, 47). However, and contrary to activated memory CD4^+^ T cells, CD26-induced IL-26^+^ CD4^+^ T cells do not express IL-2, IFNγ, and IL-17A. To date, a role for CD26 in the expression of IL-26 by effector memory T cells has not been reported.

IL-22-expressing natural killer (NK) cells, also called NK22 or innate lymphoid cell 3 (ILC3), are a NK cell subset present in mucosa-associated lymphoid tissues and involved in immune surveillance at mucosal surfaces (48). Mature NK22 cells, as well as stage 3 human immature CD34^−^ CD117^+^ CD161^+^ CD94^−^ CD56^−^ NKp44^−^ NK cells, which may constitute precursors of mature NK22 cells (49), constitutively express IL-26 mRNA (50–52). In line with this observation, IL-22-expressing, and IL-26-producing CD4^+^ T cells have been detected within skin chronic wounds due to *Staphylococcus aureus* (53).

#### Expression by Monocytes/Macrophages

Contradictory results are reported in the literature concerning the expression of IL-26 by monocytes. Although Wolk et al. showed that IL-26 is not expressed by monocytes (42, 54), other authors reported that monocytes constitutively express IL-26 mRNA, although at a low level compared to memory T lymphocytes (55). Similar contrasting results are reported on its expression by activated monocytes. IL-26 mRNA expression appears down-regulated in monocytes infected by *Mycobacterium tuberculosis* (55) while, in contrast, a stimulation with LPS plus IFNγ, in the presence of a neutralizing anti-IL-10 Ab, induces its secretion (56). Furthermore, lung alveolar macrophages from healthy volunteers secrete IL-26 after local exposure to endotoxin (25).

#### Expression by Non-immune Cells

Whereas IL-26 emerges as a mediator potentially involved in the control of tissue homeostasis, only a few studies have reported its expression by epithelial cells. Nevertheless, it has been shown that the synthetic TLR3 agonist poly[I:C] acts synergistically with IL-17A to induce IL-26 expression in primary bronchial epithelial cells (57). The expression of IL-26 was confirmed in bronchial brush biopsies from healthy subjects (57).

IL-26 has been also detected in the joints of patients suffering from rheumatoid arthritis (RA) (29) and spondyloarthritis (58). More precisely, IL-26 is expressed by fibroblast-like synoviocytes and, to a lower extent, by CD68^+^ macrophage-like synoviocytes present in inflamed joints from RA patients. The expression of IL-26 by fibroblast-like synoviocytes is potentiated by the inflammatory cytokines IL-1β and IL-17A (29). A recent study confirms the expression of IL-26 by α smooth muscle actin-expressing myofibroblasts in spondyloarthritis patients (58). We also reported the *in vitro* expression of IL-26 by primary smooth muscular cells (SMC), which is enhanced upon stimulation with IL-1β and TNFα (19). IL-26 is also detected in renal arterial SMC in patients with anti-neutrophil cytoplasmic antibodies (ANCA)-associated vasculitis (AAV) (19).

## Biological Properties of IL-26

**Figure 2:**
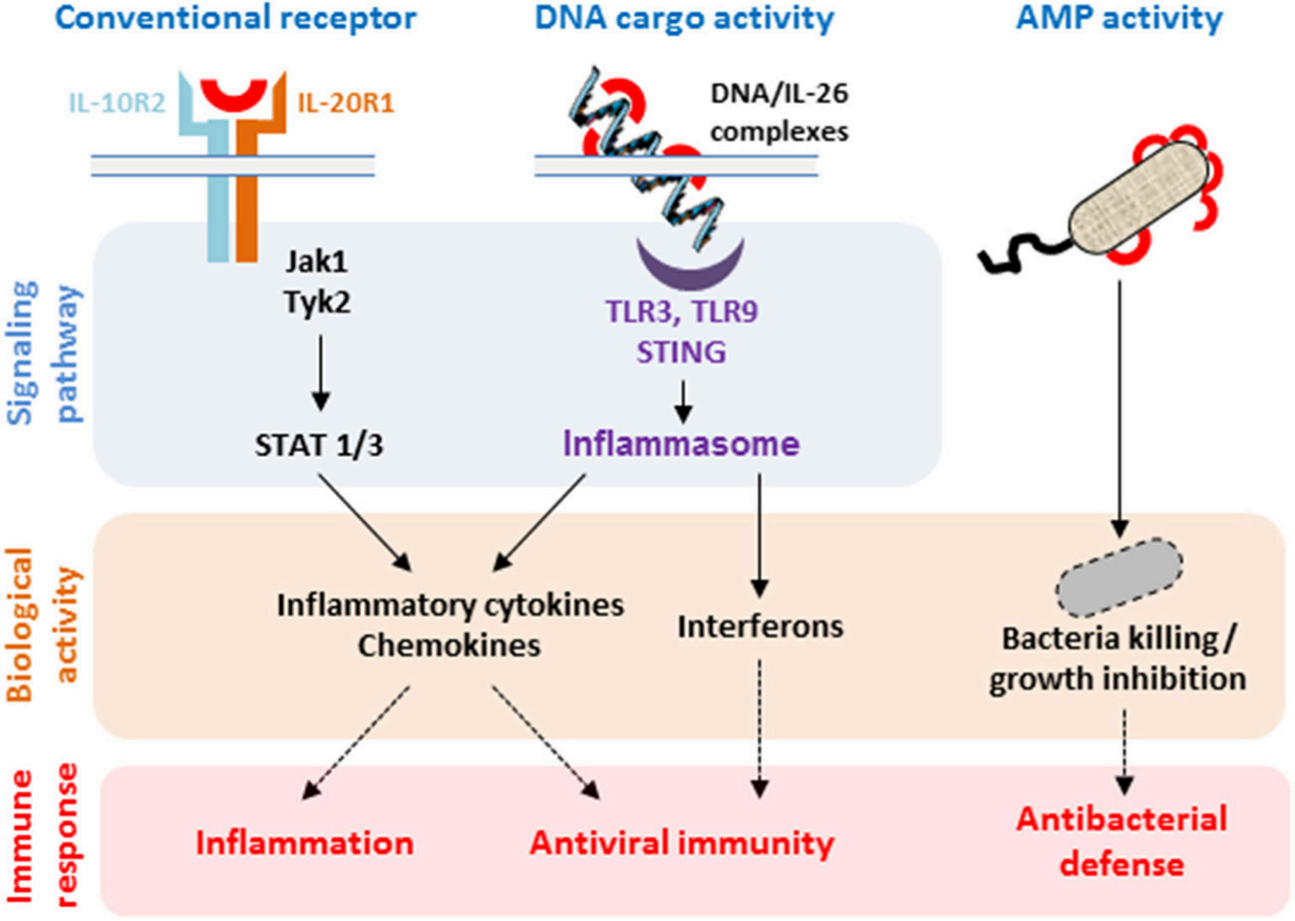
Biological properties of IL-26. The binding of IL-26 to the conventional receptor composed of the IL-20R1 and IL-10R2 chains induces the production of inflammatory cytokines. IL-26 can also act as a carrier molecule allowing extracellular DNA to get access to intracellular nucleic sensors. Both pathways induce the production of inflammatory cytokines, chemokines, and type I and type II interferons by selected immune and non-immune cells. IL-26 also acts as an antimicrobial protein (AMP) through its capacity to form pores in bacterial membranes.

Due to its absence in mice and rat, most of the biological properties of IL-26 have been described *ex vivo* using human cells (12, 13), mainly in inflammatory backgrounds (27, 29, 35, 59). Recent important studies have demonstrated that IL-26 is not strictly a cytokine but can also act as a carrier for extracellular DNA (18, 19) and as an antimicrobial molecule (18), thanks to its particular biochemical properties. These properties (Figure 2) suggest that IL-26 can be categorized as a kinocidin, an emerging family of proteins involved in intercellular dialog and sharing bactericidal properties (60–62).

### Effects on Non-immune Cells

Based on the original observation of an elevated expression of IL-26 in Crohn's disease (35), the authors reported that IL-26 induces the expression of the pro-inflammatory cytokines IL-8 and TNFα as well as the regulatory cytokine IL-10 in human gut epithelial cells (35). This activation of epithelial cells is mediated via the conventional IL-26R and involves STAT1 and/or STAT3 (24, 35). The capacity of IL-26 to stimulate epithelial cells was confirmed in a study reporting that IL-26 induces the secretion of inflammatory cytokines by the keratinocyte cell line HaCat and, to a lower extent, by human primary keratinocytes (24). In contrast to other members of the IL-10 cytokine family, IL-26 does not induce the proliferation of primary human keratinocytes, but does induce STAT3 phosphorylation in these IL-26R-expressing cells (26); however, the expression of inflammatory molecules was not monitored in this study.

In response to soluble mediators secreted by IL-26-expressing ILC3 cells, epithelial cells produce IL-10, and express a variety of mitogenic and anti-apoptotic molecules (50); however, the role of IL-26 in this process remains to be elucidated.

It is important to note that most of the above results were obtained using transformed cell lines and that a variety of other cell types, originating from pancreas, colon, cervix, skin, bone marrow or liver, do not respond to IL-26 (24). Moreover, contrasting results have also been reported using primary epithelial cells. As an example, IL-26 inhibits the production of IL-1β, TNFα, IL-8, and GM-CSF by primary bronchial epithelial cells (25) and a recent study reports that IL-26, as well as the inflammatory cytokines IL-17 and IL-22, affect the paracellular permeability in an *in vitro* model of respiratory epithelial barrier using primary human nasal epithelial cells (63). Similarly, IL-26 inhibits the proliferation of intestinal epithelial cells (35), while, in contrast, it promotes the growth of gastric tumor cells (64).

### Effects on Immune Cells

Several studies have reported that IL-26 induces the production of pro-inflammatory cytokines by innate immune cells.

#### Myeloid Cells

IL-26 induces the production of the pro-inflammatory cytokines IL-1β, IL-6, and TNFα by human monocytes (29). In agreement with the fact that IL-1β is pivotal in the generation of human Th17 cells (65), IL-26-treated monocytes induce the differentiation of non-committed memory CD4^+^ T cells into Th17 lymphocytes (29). Interestingly, IL-26-induced Th17 cells express IL-26, allowing initiating of an amplification loop between Th17 cell polarization, and inflammatory cytokine secretion that could participate in the maintenance of a pool of activated Th17 cells. IL-26 also induces human monocytes to express numerous chemokines involved in the recruitment of innate and adaptive immune cells (29). Che et al. confirmed these results by showing that IL-26 induces the release of the neutrophil-mobilizing cytokines IL-1β, TNFα, and GM-CSF by primary immune cells isolated from endotoxin-primed human airways (25).

Monocytes are precursors of macrophages, dendritic cells, and osteoblasts, highly specialized tissue-resident cells whose generation is driven by local signals. Osteoblasts and osteoclasts, which are involved in bone remodeling, are responsive to IL-26. IL-26 inhibits the bone-resorbing activity of mature osteoclasts (66) and increases bone mineralization by human osteoblasts (58).

IL-26 also primes neutrophils to IL-8- and fMLP-induced chemotaxis (but not chemokinesis) and to anti-bacterial activity (25). The capacity of neutrophils to respond to IL-26 has been confirmed *in vivo*. Indeed, an intranasal instillation of recombinant human IL-26 facilitates the recruitment of innate immune cells toward the bronchoalveolar space after a local stimulation with LPS (25, 57).

#### Lymphoid Cells

IL-26 triggers NK cell activation, as evidenced by their induced expression of the inflammatory cytokines IL-1β and TNFα. Importantly, IL-26 also promotes the production of type I (IFNβ) and type II (IFNγ) interferons (27), that induce and orchestrate innate and adaptive antiviral responses. Consistent with this observation, IL-26-treated human NK cells express elevated levels of membrane-anchored TRAIL, allowing them to kill hepatitis C virus (HCV)-infected hepatocytes (27). Whether the activation of IL-26R-negative NK cells by IL-26 is also dependent on its capacity to shuttle extracellular DNA within lymphoid cells remains to be determined.

The capacity of IL-26 to act on adaptive immune cells remains to be clarified. Hummelshoj et al. have reported that IL-26 inhibits the production of IgA and IgG by B lymphocytes activated by an anti-CD40 Ab and the production of IgG4 induced by anti-CD40 Ab plus IL-4 (28). To date, no direct effect of IL-26 on naive and memory human T cells has been reported.

#### Other Immune Cells

Plasmacytoid DC have a central role in the antiviral arsenal, thanks to their capacity to produce important amounts of IFNα (67). When combined with DNA, IL-26 induces the production of IFNα by human pDC (18).

### Antimicrobial Properties

IL-26 exhibits a direct antimicrobial activity, a property related to its biochemical, and structural similarities with antimicrobial peptides (AMP) (18, 19).

#### Antiviral Activity

Braum et al. reported for the first time a biological activity of IL-26 which is not dependent on cell activation, by showing that IL-26 modulates the rate of cell infection by enveloped viruses (31). More precisely, a pre-incubation of vesicular stomatitis virus (*VSV*) with IL-26 increases the rate of infection of the epithelial cell line Colo-205, whereas an opposite effect is observed with the human *Cytomegalovirus* (*CMV*). In contrast, IL-26 does not modulate the infection of the epithelial cell line Vero by *Herpes virus simplex 1* (*HSV-1*). Interestingly, the capacity of IL-26 to modulate viral infectivity appears independent of the expression of IL-26R (31). Even though the mechanism involved has not been elucidated, the authors suggested that the biochemical properties of IL-26 may be responsible, at least in part, for the protective activity of IL-26. This hypothesis is supported by previous studies showing that AMP can prevent virus infectivity by (i) causing virus membrane instability, rendering them unable to infect host cells (68, 69), or (ii) by interfering with the virus binding to host cells (through interaction with binding structures of the virus or with receptors at the surface of target cells) (70–72). A role for IL-26 in antiviral defense is also supported by its overexpression in the embryonic zebra fish cell line ZF4 infected by the aquatic *Birnavirus Infectious pancreatic necrosis virus* (73). AMP can also kill some enveloped viruses (74, 75). Whether IL-26 may also kill enveloped virus, in a way similar to AMP, remains unknown.

#### Antibacterial Activity

In 2015, the group of M. Gilliet reported that IL-26 acts as an antibacterial molecule (18). The initial hypothesis for the mechanism of this action was based on the particular distribution of charges at the surface of IL-26, similar to AMP. IL-26 kills or inhibits the growth of a variety of Gram positive (*Staphyloccocus aureus*) and Gram negative bacteria (*Pseudomonas aeruginosa, Escherichia coli, Klebsiella pneumoniae*) at concentrations similar to the antimicrobial molecule LL-37. The antimicrobial activity of IL-26 relies on its capacity to form pores and to disrupt bacterial membranes, as indicated by the presence of membrane blebs on the surface of the bacteria and cytosolic leakage pores in bacterial walls.

The antibacterial activity of IL-26 was also suspected to rely on its capacity to organize into multimers, thanks to the absence of a conserved proline residue (P113) which is involved in the formation of arm-exchange dimers in the other members of the IL-10 cytokine family. The authors also identified lipopolysaccharide and lipoteichoic acid as IL-26-binding elements at the surface of bacteria (18). Interestingly, the biological activity of IL-26 present in T cell culture supernatants was superior to that of recombinant proteins produced in prokaryotes (18). Post-translational modifications and/or multimer organization have been proposed to account for the superior activity of natural IL-26 compared to recombinant IL-26. Interestingly, the authors confirmed the antimicrobial activity of IL-26 in an *in vivo* model of sepsis (18).

The antimicrobial activity of IL-26 was recently confirmed by Woetmann et al. who showed that it triggers *Staphylococcus aureus* death and inhibits biofilm formation (53). In contrast, IL-26 does not affect the growth of *Propionibacterium acnes* (76).

IL-26 has also an indirect antimicrobial activity via inducing the production of type I and type II interferons by pDC, monocytes, and NK cells (18, 19, 27). Interestingly, IL-26 seems to be necessary for pDC activation, as killed *Pseudomonas* aeruginosa alone is unable to induce IFNα production (18).

These biological activities allow categorization of IL-26 as a membrane-active AMP (77, 78).

## Interleukin-26 in Human Diseases

Deciphering the biology of IL-26 was hampered by the absence of the IL26 gene in mice and rat. As initial studies revealed that activated memory Th17 cells are the main producers of IL-26 (35, 45), its properties were mostly examined in the context of chronic inflammatory and infectious diseases.

Collectively, these studies identified IL-26 as a master regulator of inflammation. Even though IL-26 can signal via the heteroduplex IL-20R1–IL-10R2 (12, 13), accumulating data suggests that the proinflammatory activity of IL-26 may rely on its capacity to form complexes with extracellular DNA (18, 19) in a context of chronic and severe inflammation associated with cell death and tissue destruction. Indeed, extracellular nucleic acids, released by damaged cells, have emerged in the recent past as major inflammation driving signals (79). It is important to note that extracellular DNA has to complex with shuttling molecules to get access to intracellular DNA sensors, underlining the role of carrier molecules in rendering extracellular DNA pro-inflammatory.

**Figure 3:**
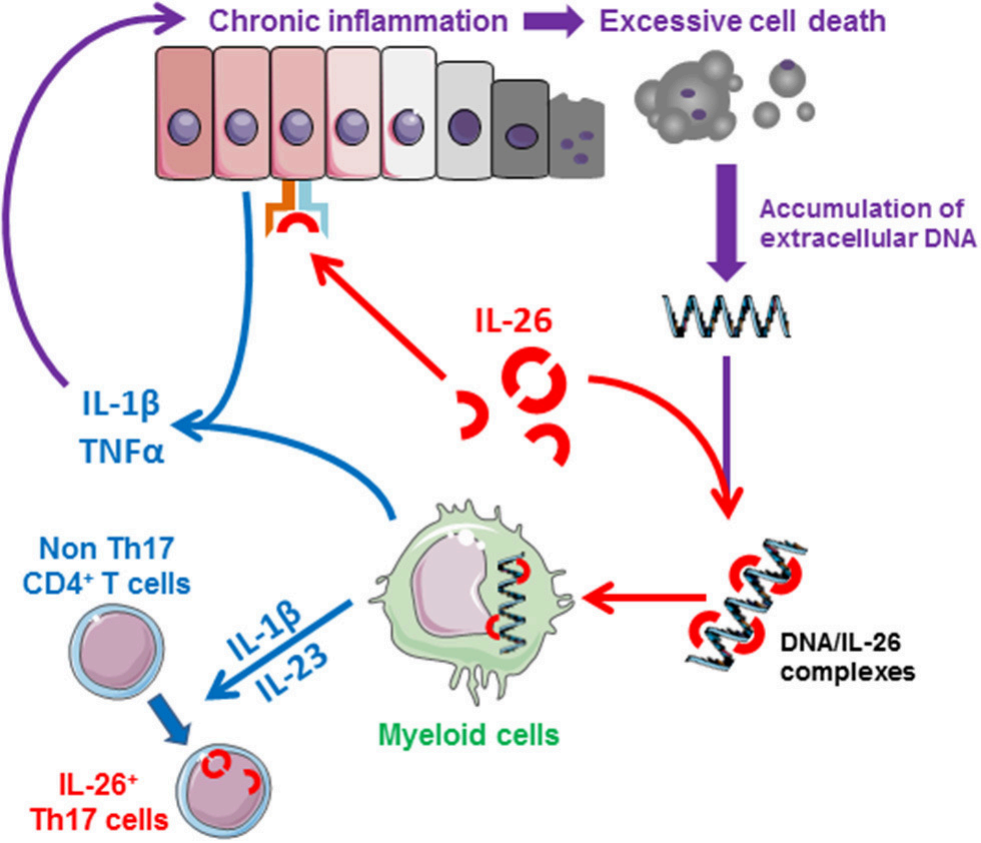
Schematic representation of the proinflammatory properties of IL-26. IL-26, produced by activated memory Th17 cells and NK22 cells, induces the production of inflammatory mediators by epithelial cells and favors the generation of inflammatory Th17 cells (induction of IL-1β and IL-23 by monocytes). In case of chronic inflammation, IL-26 can bind to extracellular DNA released by dying cells into the extracellular milieu (at sites of extensive tissue damaged). The IL-26-DNA complexes can then activate innate immune cells (such as monocytes), initiating a deleterious amplification loop leading to chronic inflammation, and tissue damages.

Importantly, recent studies also suggest that IL-26 may act as a dangerous catalyst that could contribute to the establishment of chronic inflammation. Indeed, the production of IL-26 is induced by inflammatory cytokines which, conversely, induces the production of inflammatory mediators. This mechanism may establish a deleterious positive feedback loop (i.e., production of IL-26 by activated Th17 cells and release of DNA by dying cells as a result of chronic tissue damage) leading to unabated inflammation after elimination of the initiating signal (Figure 3). The biological properties of IL-26 and related roles in human diseases are summarized in Table 1.

**Table 1:** Biological properties of IL-26 and related human diseases.

	**Target**	**Impact**	**Mechanism of action**	**Related human diseases**	**References**
Cell activation	Non immune cells Epithelial cells	Production of inflammatory cytokines Inhibition of proliferation	IL-10R2—IL-20R1	IBD, Crohn's disease Psoriasis	(24, 35, 80–83)
	Non immune cells Fibroblasts	Collagen synthesis	IL-10R2 – IL-20R1	GVHD (bronchiolitis obliterans)	(39)
	Myeloid cells	Production of inflammatory cytokines and of type I IFN	Carrier of extracellular DNA STING & inflammasome	Spondyloarthritis, RA Allergic contact dermatitis ANCA-associated vasculitis	(18, 19, 29, 58, 84)
	Lymphoid cells	Production of IFNγ Enhanced NK cell cytotoxicity	Unknown	Chronic HCV infection	(27)
	Plasmacytoid cells	Production of inflammatory cytokines and of type I IFN	Carrier of extracellular DNA TLR9	Sepsis	(18)
Antimicrobial activity	Virus	Modulation of viral replication (inhibition or activation)	Suspected modulation of virus infectivity	–	(31)
	Bacteria	Bacteria killing Growth inhibition	Formation of pores	Sepsis IBD	(18, 85)

*IBD, inflammatory bowel disease; GVHD, graft-vs.-host disease; HCV, hepatitis C virus; RA, rheumatoid arthritis*.

### Chronic Viral Infection

The levels of circulating IL-26 are increased in *HCV*-infected patients and are correlated with the inflammation stage of the liver (27). IL-26 is expressed by circulating CD4^+^ T cells and infiltrating liver T cells. It also accumulates in hepatocytes from chronically infected HCV-infected patients, although the mechanism involved remains undetermined as infected hepatocytes do not express IL-26 mRNA. Inflammation is part of the antiviral immune response; the pro-inflammatory and NK cell-stimulatory activities of IL-26 may also contribute to the control and/or elimination of virus-infected cells (27).

### Inflammatory Disorders

Several genetic studies have suggested a potential role for IL-26 in the pathophysiology of chronic inflammatory and autoimmune disorders. *IL26* gene polymorphisms are associated with an increased risk for developing multiple sclerosis (MS) (86) or RA (15). Moreover, elevated levels of circulating and tissue-associated IL-26 are reported in Th17 cell-mediated autoimmune diseases, such as Crohn's disease, RA, psoriasis, and AAV.

#### Inflammatory Bowel Diseases

The role of IL-26 in IBD was first suggested by a genetic association study which identified rs2870946 polymorphism as a strong predictive factor (80).

The levels of circulating IL-26 are elevated in patients with Crohn's disease and an increased expression of IL-26 mRNA is observed within inflammatory colonic lesions (24, 35, 81). The expression of the transcript encoding IL-26 is also increased in the colonic lesions of pediatric-onset ulcerative colitis (82). The expression of Th17 pathway-associated genes, including IL-26, is enhanced in inflamed colonic samples from IBD patients compared to controls, and their levels are correlated with disease severity (83). More specifically, in Crohn's disease, RORγt^+^ Th17 cells infiltrating inflamed mucosa secrete IL-26 which can, in turn, induce the secretion of inflammatory cytokines by epithelial cells (35).

In patients with common variable immune deficiency associated with inflammatory complications, IL-22^+^ IL-26^+^ NK22 cells are expanded in the blood and infiltrate gastrointestinal, and lung tissues (51). Pinero et al. reported that peripheral blood mononuclear cells from Crohn's disease patients with a variant IL26 genotype (varIL26) have a reduced capacity to kill bacteria compared to wild type IL26-genotyped (wtIL26) patients and that the varIL26 genotype is associated with reduced IL-26 serum levels (87). Although Crohn's disease patients with wtIL26 or varIL26 genotypes exhibit similar rates of circulating bacterial DNA, the concentrations of IFNγ, IL-12, and TNFα are elevated in patients with a varIL26 genotype and containing circulating bacteria DNA. The authors suggest that IL26 polymorphisms may impact bacterial DNA clearance and, consequently, the amplitude and duration of inflammatory responses in IBD patients (87).

#### Rheumatoid Arthritis

The levels of IL-26 are increased in the sera and synovial fluids of RA patients, compared to healthy subjects. Moreover, in RA patients, the levels of IL-26 are higher in synovial fluids than in the serum, suggesting a local production in inflamed joints (29).

The role of IL-26 in inflammatory articular diseases has been recently confirmed in a study showing that the levels of IL-26 are increased in the synovial fluids of patients suffering from spondyloarthritis (58). However, IL-26 does not appear directly involved in the bone destruction process, as it suppresses RANKL-induced osteoclastogenesis (66).

#### Vasculitis

ANCA-associated vasculitis is a chronic relapsing autoimmune inflammatory disease associated with extensive cell death. Acute flares are characterized by necrotic lesions in microvessels where the massive accumulation of dying neutrophils induces the subsequent recruitment and activation of immune cells, especially in granulomatous lesions (88, 89). The inflammatory process is suspected to result from the release of neutrophil self DNA into the extracellular milieu (90).

Patients with active AAV have high levels of both circulating IL-26 and IL-26-DNA complexes, especially during acute flares (19). Moreover, in patients with rapidly progressive glomerulonephritis, IL-26 deposits in necrotizing lesions (19). These data support our hypothesis that IL-26-DNA complexes expressed in inflammatory lesions may initiate a positive amplification loop between extensive cell death and chronic inflammation.

Increased levels of IL-26 are also detected in the sera of patients suffering from Behçet's disease, a rare auto-inflammatory disorder with manifestations of vasculitis; in this context, IL-26 levels correlate with disease severity and it accumulates in inflammatory lesions (46, 91).

#### Other Chronic Inflammatory Diseases

An accumulation of IL-26 has been reported in psoriasis lesions (18, 45). IL-26 mRNA and protein were also evidenced in early primary systemic sclerosis skin lesions and its expression was positively correlated with the disease activity score (37). However, the role of IL-26 in the pathophysiology of this disease remains to be investigated.

Although the levels of IL-26 were reported elevated in MS patients treated with IFNβ (92), a recent study did not find any statistically significant modulation of IL-26 mRNA expression between healthy controls and stable or relapsing MS patients (93).

### Graft vs. Host Disease

Chronic inflammation can lead to fibrosis (94). However, the potential role of IL-26 in this process has been only evoked in a study using a mouse model of pulmonary chronic GVHD (95). The authors showed that sub-lethally irradiated NOD/Shi-scidIL2rg^null^ mice transplanted with human umbilical cord blood develop symptoms of chronic GVHD, such as bronchiolitis obliterans, scleroderma, and primary biliary cirrhosis-like lesions. IL-26 increases collagen synthesis by fibroblasts, promoting lung fibrosis during GVHD induced after the adoptive transfer of IL-26^+^ CD4^+^ T cells to IL-26-transgenic mice. The key role of IL-26 was reinforced by the demonstration that a neutralizing anti-IL-26 Ab decreased collagen deposition in pulmonary chronic GVHD in mice.

In humans, immunohistochemistry detected an infiltration of IL-26^+^ CD26^+^ CD4^+^ T cells within bronchial fibrotic lesions in a patient suffering from bronchiolitis obliterans after allogenic hematopoietic stem cell transplantation (39, 47).

### Allergy

A single nucleotide polymorphism (rs3741809) in the *IL26* gene is associated with susceptibility to allergic rhinitis (96) and the levels of IL-26 in sputum samples are positively correlated with pediatric asthma severity (17). More recently, the role of IL-26 in allergic contact dermatitis (ACD) has been investigated. In this disease, skin injuries have been assumed to be related to T cell-mediated cytotoxicity against keratinocytes. The expression of IL-26 is increased in the sera and in the skin of ACD patients (elevated IL-26 mRNA expression and infiltration of IL-26-expressing cells in skin lesions), compared to healthy subjects. *In vitro*, peripheral blood mononuclear cells (PBMC) isolated from ACD patients have a higher cytotoxic activity against the keratinocyte cell line HaCat compared to PBMC from healthy subjects. Interestingly, the cytotoxic activity was strongly decreased after silencing IL-26 mRNA expression (84).

### Cancer

IL-26 mRNA is highly expressed in cutaneous biopsies of patients with primary cutaneous T-cell lymphomas (CTCL), at levels similar to patients with psoriasis (97). Many cancers may arise from sites of infection and/or chronic irritation and inflammation. Moreover, inflammation modulates neoplastic processes, proliferation, and migration of tumor cells. As IL-26 is a pro-inflammatory cytokine, one can suspect that it may also participate in tumor initiation and/or progression. Studies are needed to clarify this important question.

## Conclusion

IL-26 has emerged as an inflammatory mediator acting upstream in the inflammation cascade. Nevertheless, and contrary to other members of the IL-10 family cytokines, the pro-inflammatory properties of IL-26 appear mainly related to its ability to act as a carrier of extracellular DNA, a property that would be most important during chronic inflammation which is associated with tissue damage. This unique property identifies IL-26 as a master driver of chronic inflammation.

IL-26 also exhibits antibacterial properties, thanks to its particular structural similarities to AMP.

Importantly, most of the biological functions of IL-26 have been identified in pathological situations that feature chronic inflammation. Thus, the roles of IL-26 in normal physiology remain largely unknown. One can hypothesize that IL-26 could be involved in mucosal innate immunity, as suggested by its expression in colon, ileum, Payer's patches, and tonsils. Deciphering its physiological properties is of importance, notably since neutralizing IL-26 has been proposed as a novel therapeutic strategy for some Th17-associated disorders.

In light of recent studies, it becomes evident that IL-26 is more than a cytokine. The dual ability to act as a cytokine and as an AMP allows classification of IL-26 in the kinocidin family. Nevertheless, our knowledge of the biology of IL-26 remains fragmentary and future studies are required to decipher the biology of this amazing molecule.

## Author Contributions

VL, CM, CP, EB, PR, HF, PJ, and YD defined the content of the manuscript and contributed to manuscript writing. VL and YD created graphical illustrations. All authors approved the final version of the manuscript.

### Conflict of Interest Statement

The authors declare that the research was conducted in the absence of any commercial or financial relationships that could be construed as a potential conflict of interest.

## Abbreviations

AAV: ANCA-associated vasculitis
Ab: antibody
ACD: allergy contact dermatitis
AMP: antimicrobial peptide
ANCA: anti-neutrophil cytoplasmic antibodies
CMV: cytomegalovirus
CPP: cell-penetrating peptide
CRF: cytokine receptor family
CTCL: cutaneous T cell lymphoma
DC: dendritic cells
GVHD: graft-vs. -host disease
HSV-1: herpes simplex virus 1
HVS: herpesvirus saimiri
IBD: inflammatory bowel disease
IFN: interferon
IL: interleukin
ILC: innate lymphoid cell
IPM: in-plane membrane
Jak: janus kinase
MS: multiple sclerosis
NK: natural killer
pDC: plasmacytoid dendritic cells
RA: rheumatoid arthritis
RANKL: receptor activator of nuclear factor kappa-B ligand
SMC: smooth muscle cell
STAT: signal transducer and activator of transcription
STING: stimulator of interferon genes
TLR: toll-like receptor
TNF: tumor necrosis factor
Treg: regulatory T lymphocytes
Tyk: tyrosine kinase
VSV: vesicular stomatitis virus.

## References

[1] Schwartz DM Bonelli M Gadina M O'Shea JJ. Type I/II cytokines, JAKs, and new strategies for treating autoimmune diseases. Nat Rev Rheumatol. (2016) 12:25–36. 10.1038/nrrheum.2015.167 26633291 PMC4688091

[2] Dinarello CA. Introduction to the interleukin-1 family of cytokines and receptors: drivers of innate inflammation and acquired immunity. Immunol Rev. (2018) 281:5–7. 10.1111/imr.12624 29248001 PMC5750395

[3] Fujio K Komai T Inoue M Morita K Okamura T Yamamoto K. Revisiting the regulatory roles of the TGF-beta family of cytokines. Autoimmun Rev. (2016) 15:917–22. 10.1016/j.autrev.2016.07.007 27392504

[4] Croft M Siegel RM. Beyond TNF: TNF superfamily cytokines as targets for the treatment of rheumatic diseases. Nat Rev Rheumatol. (2017) 13:217–33. 10.1038/nrrheum.2017.22 28275260 PMC5486401

[5] Choura M Rebai A. Receptor tyrosine kinases: from biology to pathology. J Recept Signal Transduct Res. (2011) 31:387–94. 10.3109/10799893.2011.625425 22040163

[6] Griffith JW Sokol CL Luster AD. Chemokines and chemokine receptors: positioning cells for host defense and immunity. Annu Rev Immunol. (2014) 32:659–702. 10.1146/annurev-immunol-032713-120145 24655300

[7] Akdis M Aab A Altunbulakli C Azkur K Costa RA Crameri R. Interleukins (from IL-1 to IL-38), interferons, transforming growth factor beta, and TNF-alpha: receptors, functions, and roles in diseases. J Allergy Clin Immunol. (2016) 138:984–1010. 10.1016/j.jaci.2016.06.033 27577879

[8] Brocker C Thompson D Matsumoto A Nebert DW Vasiliou V. Evolutionary divergence and functions of the human interleukin (IL) gene family. Hum Genomics (2010) 5:30–55. 10.1186/1479-7364-5-1-30 21106488 PMC3390169

[9] Ouyang W Rutz S Crellin NK Valdez PA Hymowitz SG. Regulation and functions of the IL-10 family of cytokines in inflammation and disease. Annu Rev Immunol. (2011) 29:71–109. 10.1146/annurev-immunol-031210-101312 21166540

[10] Rutz S Eidenschenk C Ouyang W IL-22, not simply a Th17 cytokine. Immunol Rev. (2013) 252:116–32. 10.1111/imr.12027 23405899

[11] Knappe A Hor S Wittmann S Fickenscher H. Induction of a novel cellular homolog of interleukin-10, AK155, by transformation of T lymphocytes with herpesvirus saimiri. J Virol. (2000) 74:3881–7. 10.1128/JVI.74.8.3881-3887.2000 10729163 PMC111897

[12] Donnelly RP Sheikh F Dickensheets H Savan R Young HA Walter MR. Interleukin-26: an IL-10-related cytokine produced by Th17 cells. Cytokine Growth Factor Rev. (2010) 21:393–401. 10.1016/j.cytogfr.2010.09.001 20947410 PMC2997847

[13] Stephen-Victor E Fickenscher H Bayry J. IL-26: an emerging proinflammatory member of the IL-10 cytokine family with multifaceted actions in antiviral, antimicrobial, and autoimmune responses. PLoS Pathog. (2016) 12:e1005624. 10.1371/journal.ppat.1005624 27337042 PMC4919036

[14] Lutfalla G Roest Crollius H Stange-Thomann N Jaillon O Mogensen K Monneron D. Comparative genomic analysis reveals independent expansion of a lineage-specific gene family in vertebrates: the class II cytokine receptors and their ligands in mammals and fish. BMC Genomics (2003) 4:29. 10.1186/1471-2164-4-29 12869211 PMC179897

[15] Vandenbroeck K Cunningham S Goris A Alloza I Heggarty S Graham C. Polymorphisms in the interferon-gamma/interleukin-26 gene region contribute to sex bias in susceptibility to rheumatoid arthritis. Arthritis Rheum. (2003) 48:2773–8. 10.1002/art.11236 14558082

[16] Goris A Marrosu MG Vandenbroeck K. Novel polymorphisms in the IL-10 related AK155 gene (chromosome 12q15). Genes Immun. (2001) 2:284–6. 10.1038/sj.gene.6363772 11528524

[17] Collins PL Henderson MA Aune TM. Lineage-specific adjacent IFNG and IL26 genes share a common distal enhancer element. Genes Immun. (2012) 13:481–8. 10.1038/gene.2012.22 22622197 PMC4180225

[18] Meller S Di Domizio J Voo KS Friedrich HC Chamilos G Ganguly D. T(H)17 cells promote microbial killing and innate immune sensing of DNA via interleukin 26. Nat Immunol. (2015) 16:970–9. 10.1038/ni.3211 26168081 PMC4776746

[19] Poli C Augusto JF Dauve J Adam C Preisser L Larochette V. IL-26 Confers Proinflammatory properties to extracellular DNA. J Immunol. (2017) 198:3650–61. 10.4049/jimmunol.1600594 28356384

[20] Copolovici DM Langel K Eriste E Langel U. Cell-penetrating peptides: design, synthesis, and applications. ACS Nano (2014) 8:1972–94. 10.1021/nn4057269 24559246

[21] Choi YS David AE. Cell penetrating peptides and the mechanisms for intracellular entry. Curr Pharm Biotechnol. (2014) 15:192–9. 10.2174/1389201015666140617093331 24938895

[22] Sabat R. IL-10 family of cytokines. Cytokine Growth Factor Rev. (2010) 21:315–24. 10.1016/j.cytogfr.2010.11.001 21112807

[23] Sheikh F Baurin VV Lewis-Antes A Shah NK Smirnov SV Anantha S. Cutting edge: IL-26 signals through a novel receptor complex composed of IL-20 receptor 1 and IL-10 receptor 2. J Immunol. (2004) 172:2006–10. 10.4049/jimmunol.172.4.2006 14764663

[24] Hor S Pirzer H Dumoutier L Bauer F Wittmann S Sticht H. The T-cell lymphokine interleukin-26 targets epithelial cells through the interleukin-20 receptor 1 and interleukin-10 receptor 2 chains. J Biol Chem. (2004) 279:33343–51. 10.1074/jbc.M405000200 15178681

[25] Che KF Tengvall S Levanen B Silverpil E Smith ME Awad M. Interleukin-26 in antibacterial host defense of human lungs. Effects on neutrophil mobilization. Am J Respir Crit Care Med. (2014) 190:1022–31. 10.1164/rccm.201404-0689OC 25291379

[26] Gough P Ganesan S Datta SK. IL-20 signaling in activated human neutrophils inhibits neutrophil migration and function. J Immunol. (2017) 198:4373–82. 10.4049/jimmunol.1700253 28424238 PMC5476316

[27] Miot C Beaumont E Duluc D Le Guillou-Guillemette H Preisser L Garo E. IL-26 is overexpressed in chronically HCV-infected patients and enhances TRAIL-mediated cytotoxicity and interferon production by human NK cells. Gut (2015) 64:1466–75. 10.1136/gutjnl-2013-306604 25183206

[28] Hummelshoj L Ryder LP Poulsen LK. The role of the interleukin-10 subfamily members in immunoglobulin production by human B cells. Scand J Immunol. (2006) 64:40–7. 10.1111/j.1365-3083.2006.01773.x 16784489

[29] Corvaisier M Delneste Y Jeanvoine H Preisser L Blanchard S Garo E. IL-26 is overexpressed in rheumatoid arthritis and induces proinflammatory cytokine production and Th17 cell generation. PLoS Biol. (2012) 10:e1001395. 10.1371/journal.pbio.1001395 23055831 PMC3463509

[30] Che KF Tufvesson E Tengvall S Lappi-Blanco E Kaarteenaho R Levanen B. The neutrophil-mobilizing cytokine interleukin-26 in the airways of long-term tobacco smokers. Clin Sci. (2018) 132:959–83. 10.1042/CS20180057 29780024 PMC6365630

[31] Braum O Klages M Fickenscher H. The cationic cytokine IL-26 differentially modulates virus infection in culture. PLoS ONE (2013) 8:e70281. 10.1371/journal.pone.0070281 23875025 PMC3707906

[32] Sapay N Guermeur Y Deleage G. Prediction of amphipathic in-plane membrane anchors in monotopic proteins using a SVM classifier. BMC Bioinformatics (2006) 7:255. 10.1186/1471-2105-7-255 16704727 PMC1564421

[33] Avci FG Akbulut BS Ozkirimli E. Membrane active peptides and their biophysical characterization. Biomolecules (2018) 8:E77. 10.3390/biom8030077 30135402 PMC6164437

[34] Tengvall S Che KF Linden A. Interleukin-26: an emerging player in host defense and inflammation. J Innate Immun. (2016) 8:15–22. 10.1159/000434646 26202572 PMC6738771

[35] Dambacher J Beigel F Zitzmann K De Toni EN Goke B Diepolder HM. The role of the novel Th17 cytokine IL-26 in intestinal inflammation. Gut (2009) 58:1207–17. 10.1136/gut.2007.130112 18483078

[36] Pene J Chevalier S Preisser L Venereau E Guilleux MH Ghannam S. Chronically inflamed human tissues are infiltrated by highly differentiated Th17 lymphocytes. J Immunol. (2008) 180:7423–30. 10.4049/jimmunol.180.11.7423 18490742

[37] Zhou Y Hou W Xu K Han D Jiang C Mou K. The elevated expression of Th17-related cytokines and receptors is associated with skin lesion severity in early systemic sclerosis. Hum Immunol. (2015) 76:22–9. 10.1016/j.humimm.2014.12.008 25500255

[38] Konradsen JR Nordlund B Levanen B Hedlin G Linden A. The cytokine interleukin-26 as a biomarker in pediatric asthma. Respir Res. (2016) 17:32. 10.1186/s12931-016-0351-6 27029915 PMC4815075

[39] Ohnuma K Hatano R Aune TM Otsuka H Iwata S Dang NH. Regulation of pulmonary graft-versus-host disease by IL-26+CD26+CD4 T lymphocytes. J Immunol. (2015) 194:3697–712. 10.4049/jimmunol.1402785 25786689 PMC4568737

[40] Dagur PK Biancotto A Wei L Sen HN Yao M Strober W. MCAM-expressing CD4(+) T cells in peripheral blood secrete IL-17A and are significantly elevated in inflammatory autoimmune diseases. J Autoimmun. (2011) 37:319–27. 10.1016/j.jaut.2011.09.003 21959269 PMC3223259

[41] Anuradha R George PJ Hanna LE Kumaran P Chandrasekaran V Nutman TB. Expansion of parasite-specific CD4+ and CD8+ T cells expressing IL-10 superfamily cytokine members and their regulation in human lymphatic filariasis. PLoS Negl Trop Dis. (2014) 8:e2762. 10.1371/journal.pntd.0002762 24699268 PMC3974669

[42] Wolk K Kunz S Asadullah K Sabat R. Cutting edge: immune cells as sources and targets of the IL-10 family members? J Immunol. (2002) 168:5397–402. 10.4049/jimmunol.168.11.5397 12023331

[43] Chen Z O'Shea JJ. Regulation of IL-17 production in human lymphocytes. Cytokine (2008) 41:71–8. 10.1016/j.cyto.2007.09.009 17981475 PMC11225033

[44] Volpe E Touzot M Servant N Marloie-Provost MA Hupe P Barillot E. Multiparametric analysis of cytokine-driven human Th17 differentiation reveals a differential regulation of IL-17 and IL-22 production. Blood (2009) 114:3610–4. 10.1182/blood-2009-05-223768 19704117

[45] Wilson NJ Boniface K Chan JR McKenzie BS Blumenschein WM Mattson JD. Development, cytokine profile and function of human interleukin 17-producing helper T cells. Nat Immunol. (2007) 8:950–7. 10.1038/ni1497 17676044

[46] Kaabachi W Bouali E Berraies A Dhifallh IB Hamdi B Hamzaoui K. Interleukin-26 is overexpressed in Behcet's disease and enhances Th17 related -cytokines. Immunol Lett. (2017) 190:177–84. 10.1016/j.imlet.2017.08.008 28811236

[47] Ohnuma K Hatano R Itoh T Iwao N Dang NH Morimoto C. Role of IL-26+CD26+CD4 T cells in pulmonary chronic graft-versus-host disease and treatment with caveolin-1-Ig Fc conjugate. Crit Rev Immunol. (2016) 36:239–67. 10.1615/CritRevImmunol.2016018772 28008806

[48] Dudakov JA Hanash AM van den Brink MR. Interleukin-22: immunobiology and pathology. Annu Rev Immunol. (2015) 33:747–85. 10.1146/annurev-immunol-032414-112123 25706098 PMC4407497

[49] Tang Q Ahn YO Southern P Blazar BR Miller JS Verneris MR. Development of IL-22-producing NK lineage cells from umbilical cord blood hematopoietic stem cells in the absence of secondary lymphoid tissue. Blood (2011) 117:4052–5. 10.1182/blood-2010-09-303081 21310921 PMC3087531

[50] Cella M Fuchs A Vermi W Facchetti F Otero K Lennerz JK. A human natural killer cell subset provides an innate source of IL-22 for mucosal immunity. Nature (2009) 457:722–5. 10.1038/nature07537 18978771 PMC3772687

[51] Cols M Rahman A Maglione PJ Garcia-Carmona Y Simchoni N Ko HM. Expansion of inflammatory innate lymphoid cells in patients with common variable immune deficiency. J Allergy Clin Immunol. (2016) 137:1206–15 e6. 10.1016/j.jaci.2015.09.013 26542033 PMC4866594

[52] Hughes T Becknell B McClory S Briercheck E Freud AG Zhang X. Stage 3 immature human natural killer cells found in secondary lymphoid tissue constitutively and selectively express the TH 17 cytokine interleukin-22. Blood (2009) 113:4008–10. 10.1182/blood-2008-12-192443 19244159 PMC2673127

[53] Woetmann A Alhede M Dabelsteen S Bjarnsholt T Rybtke M Nastasi C. Interleukin-26 (IL-26) is a novel anti-microbial peptide produced by T cells in response to staphylococcal enterotoxin. Oncotarget (2018) 9:19481–9. 10.18632/oncotarget.24603 29731960 PMC5929403

[54] Wolk K Witte K Witte E Proesch S Schulze-Tanzil G Nasilowska K. Maturing dendritic cells are an important source of IL-29 and IL-20 that may cooperatively increase the innate immunity of keratinocytes. J Leukoc Biol. (2008) 83:1181–93. 10.1189/jlb.0807525 18281438

[55] Guerra-Laso JM Raposo-Garcia S Garcia-Garcia S Diez-Tascon C Rivero-Lezcano OM. Microarray analysis of *Mycobacterium tuberculosis*-infected monocytes reveals IL26 as a new candidate gene for tuberculosis susceptibility. Immunology (2015) 144:291–301. 10.1111/imm.12371 25157980 PMC4298423

[56] Nagalakshmi ML Murphy E McClanahan T de Waal Malefyt R. Expression patterns of IL-10 ligand and receptor gene families provide leads for biological characterization. Int Immunopharmacol. (2004) 4:577–92. 10.1016/j.intimp.2004.01.007 15120644

[57] Che KF Kaarteenaho R Lappi-Blanco E Levanen B Sun J Wheelock A. Interleukin-26 production in human primary bronchial epithelial cells in response to viral stimulation: modulation by Th17 cytokines. Mol Med. (2017) 23:247–57. 10.2119/molmed.2016.00064 28853490 PMC5653736

[58] Heftdal LD Andersen T Jaehger D Woetmann A Ostgard R Kenngott EE. Synovial cell production of IL-26 induces bone mineralization in spondyloarthritis. J Mol Med. (2017) 95:779–87. 10.1007/s00109-017-1528-2 28365787

[59] Li MC He SH. IL-10 and its related cytokines for treatment of inflammatory bowel disease. World J Gastroenterol. (2004) 10:620–5. 10.3748/wjg.v10.i5.620 14991925 PMC4716896

[60] Yeaman MR Cheng D Desai B Kupferwasser LI Xiong YQ Gank KD. Susceptibility to thrombin-induced platelet microbicidal protein is associated with increased fluconazole efficacy against experimental endocarditis due to Candida albicans. Antimicrob Agents Chemother. (2004) 48:3051–6. 10.1128/AAC.48.8.3051-3056.2004 15273120 PMC478484

[61] Yeaman MR Yount NY. Unifying themes in host defence effector polypeptides. Nat Rev Microbiol. (2007) 5:727–40. 10.1038/nrmicro1744 17703227

[62] Yeaman MR Yount NY Waring AJ Gank KD Kupferwasser D Wiese R. Modular determinants of antimicrobial activity in platelet factor-4 family kinocidins. Biochim Biophys Acta (2007) 1768:609–19. 10.1016/j.bbamem.2006.11.010 17217910 PMC2827485

[63] Fan K Ritter C Nghiem P Blom A Verhaegen ME Dlugosz A. Circulating cell-free miR-375 as surrogate marker of tumor burden in Merkel cell carcinoma. Clin Cancer Res. (2018) 24:5873–82 10.1158/1078-0432.CCR-18-1184 30061360 PMC6352975

[64] Harris PA Berger SB Jeong JU Nagilla R Bandyopadhyay D Campobasso N. Discovery of a first-in-class receptor interacting protein 1 (RIP1) kinase specific clinical candidate (GSK2982772) for the treatment of inflammatory diseases. J Med Chem. (2017) 60:1247–61. 10.1021/acs.jmedchem.6b01751 28151659

[65] Zielinski CE Mele F Aschenbrenner D Jarrossay D Ronchi F Gattorno M. Pathogen-induced human TH17 cells produce IFN-gamma or IL-10 and are regulated by IL-1beta. Nature (2012) 484:514–8. 10.1038/nature10957 22466287

[66] Peng YJ Wang CY Lin YH Lin GJ Huang SH Shyu JF. Interleukin 26 suppresses receptor activator of nuclear factor kappaB ligand induced osteoclastogenesis via down-regulation of nuclear factor of activated T-cells, cytoplasmic 1 and nuclear factor kappaB activity. Rheumatology (2016) 55:2074–83. 10.1093/rheumatology/kew302 27550297

[67] Fitzgerald-Bocarsly P Dai J Singh S. Plasmacytoid dendritic cells and type I IFN: 50 years of convergent history. Cytokine Growth Factor Rev. (2008) 19:3–19. 10.1016/j.cytogfr.2007.10.006 18248767 PMC2277216

[68] Robinson WE Jr McDougall B Tran D Selsted ME. Anti-HIV-1 activity of indolicidin, an antimicrobial peptide from neutrophils. J Leukoc Biol. (1998) 63:94–100. 10.1002/jlb.63.1.94 9469478

[69] Belaid A Aouni M Khelifa R Trabelsi A Jemmali M Hani K. *In vitro* antiviral activity of dermaseptins against herpes simplex virus type 1. J Med Virol. (2002) 66:229–34. 10.1002/jmv.2134 11782932

[70] Tamamura H Ishihara T Otaka A Murakami T Ibuka T Waki M. Analysis of the interaction of an anti-HIV peptide, T22 ([Tyr5, 12, Lys7]-polyphemusin II), with gp120 and CD4 by surface plasmon resonance. Biochim Biophys Acta (1996) 1298:37–44. 10.1016/S0167-4838(96)00113-6 8948487

[71] Song BH Lee GC Moon MS Cho YH Lee CH. Human cytomegalovirus binding to heparan sulfate proteoglycans on the cell surface and/or entry stimulates the expression of human leukocyte antigen class I. J Gen Virol. (2001) 82 (Pt 10):2405–13. 10.1099/0022-1317-82-10-2405 11562534

[72] Yasin B Wang W Pang M Cheshenko N Hong T Waring AJ. Theta defensins protect cells from infection by herpes simplex virus by inhibiting viral adhesion and entry. J Virol. (2004) 78:5147–56. 10.1128/JVI.78.10.5147-5156.2004 15113897 PMC400355

[73] Wang WL Liu W Gong HY Hong JR Lin CC Wu JL. Activation of cytokine expression occurs through the TNFalpha/NF-kappaB-mediated pathway in birnavirus-infected cells. Fish Shellfish Immunol. (2011) 31:10–21. 10.1016/j.fsi.2011.01.015 21272652

[74] Cruz J Ortiz C Guzman F Fernandez-Lafuente R Torres R. Antimicrobial peptides: promising compounds against pathogenic microorganisms. Curr Med Chem. (2014) 21:2299–321. 10.2174/0929867321666140217110155 24533812

[75] Kosciuczuk EM Lisowski P Jarczak J Strzalkowska N Jozwik A Horbanczuk J. Cathelicidins: family of antimicrobial peptides. A review. Mol Biol Rep. (2012) 39:10957–70. 10.1007/s11033-012-1997-x 23065264 PMC3487008

[76] Agak GW Kao S Ouyang K Qin M Moon D Butt A. Phenotype and antimicrobial activity of Th17 cells induced by propionibacterium acnes strains associated with healthy and acne skin. J Invest Dermatol. (2017) 138:316–24 10.1016/j.jid.2017.07.842 28864077 PMC5794628

[77] Bechinger B. The structure, dynamics and orientation of antimicrobial peptides in membranes by multidimensional solid-state NMR spectroscopy. Biochim Biophys Acta (1999) 1462:157–83. 10.1016/S0005-2736(99)00205-9 10590307

[78] Bahar AA Ren D. Antimicrobial peptides. Pharmaceuticals (2013) 6:1543–75. 10.3390/ph6121543 24287494 PMC3873676

[79] Crowl JT Gray EE Pestal K Volkman HE Stetson DB. Intracellular nucleic acid detection in autoimmunity. Annu Rev Immunol. (2017) 35:313–36. 10.1146/annurev-immunol-051116-052331 28142323 PMC6435037

[80] Silverberg MS Cho JH Rioux JD McGovern DP Wu J Annese V. Ulcerative colitis-risk loci on chromosomes 1p36 and 12q15 found by genome-wide association study. Nat Genet. (2009) 41:216–20. 10.1038/ng.275 19122664 PMC2652837

[81] Fujii M Nishida A Imaeda H Ohno M Nishino K Sakai S. Expression of Interleukin-26 is upregulated in inflammatory bowel disease. World J Gastroenterol. (2017) 23:5519–29. 10.3748/wjg.v23.i30.5519 28852311 PMC5558115

[82] Kugathasan S Baldassano RN Bradfield JP Sleiman PM Imielinski M Guthery SL. Loci on 20q13 and 21q22 are associated with pediatric-onset inflammatory bowel disease. Nat Genet. (2008) 40:1211–5. 10.1038/ng.203 18758464 PMC2770437

[83] Bogaert S Laukens D Peeters H Melis L Olievier K Boon N. Differential mucosal expression of Th17-related genes between the inflamed colon and ileum of patients with inflammatory bowel disease. BMC Immunol. (2010) 11:61. 10.1186/1471-2172-11-61 21144017 PMC3016394

[84] Caiazzo G Di Caprio R Lembo S Raimondo A Scala E Patruno C. IL-26 in allergic contact dermatitis: resource in a state of readiness. Exp Dermatol. (2018) 27:681–4. 10.1111/exd.13521 29498775

[85] Pinero P Juanola O Caparros E Zapater P Gimenez P Gonzalez-Navajas JM. Toll-like receptor polymorphisms compromise the inflammatory response against bacterial antigen translocation in cirrhosis. Sci Rep. (2017) 7:46425. 10.1038/srep46425 28418003 PMC5394473

[86] Goris A Heggarty S Marrosu MG Graham C Billiau A Vandenbroeck K. Linkage disequilibrium analysis of chromosome 12q14-15 in multiple sclerosis: delineation of a 118-kb interval around interferon-gamma (IFNG) that is involved in male versus female differential susceptibility. Genes Immun. (2002) 3:470–6. 10.1038/sj.gene.6363913 12486605

[87] Pinero P Juanola O Gutierrez A Zapater P Gimenez P Steinert A. IL26 modulates cytokine response and anti-TNF consumption in Crohn's disease patients with bacterial DNA. J Mol Med. (2017) 95:1227–36. 10.1007/s00109-017-1585-6 28879509

[88] Pagnoux C. Updates in ANCA-associated vasculitis. Eur J Rheumatol. (2016) 3:122–33. 10.5152/eurjrheum.2015.0043 27733943 PMC5058451

[89] Jarrot PA Kaplanski G. Pathogenesis of ANCA-associated vasculitis: an update. Autoimmun Rev. (2016) 15:704–13. 10.1016/j.autrev.2016.03.007 26970490

[90] Jennette JC Falk RJ. Pathogenesis of antineutrophil cytoplasmic autoantibody-mediated disease. Nat Rev Rheumatol. (2014) 10:463–73. 10.1038/nrrheum.2014.103 25003769

[91] Lopalco G Lucherini OM Lopalco A Venerito V Fabiani C Frediani B. Cytokine signatures in mucocutaneous and ocular behcet's disease. Front Immunol. (2017) 8:200. 10.3389/fimmu.2017.00200 28289419 PMC5327443

[92] Esendagli G Kurne AT Sayat G Kilic AK Guc D Karabudak R. Evaluation of Th17-related cytokines and receptors in multiple sclerosis patients under interferon beta-1 therapy. J Neuroimmunol. (2013) 255:81–4. 10.1016/j.jneuroim.2012.10.009 23177721

[93] Muls N Nasr Z Dang HA Sindic C van Pesch V. IL-22, GM-CSF and IL-17 in peripheral CD4+ T cell subpopulations during multiple sclerosis relapses and remission. Impact of corticosteroid therapy. PLoS ONE (2017) 12:e0173780. 10.1371/journal.pone.0173780 28301515 PMC5354390

[94] Ueha S Shand FH Matsushima K. Cellular and molecular mechanisms of chronic inflammation-associated organ fibrosis. Front Immunol. (2012) 3:71. 10.3389/fimmu.2012.00071 22566952 PMC3342381

[95] Sziksz E Pap D Lippai R Beres NJ Fekete A Szabo AJ. Fibrosis related inflammatory mediators: role of the IL-10 cytokine family. Mediat Inflamm. (2015) 2015:764641. 10.1155/2015/764641 26199463 PMC4495231

[96] Zhang Y Li J Wang C Zhang L. Association between the interaction of key genes involved in effector T-cell pathways and susceptibility to develop allergic rhinitis: a population-based case-control association study. PLoS ONE (2015) 10:e0131248. 10.1371/journal.pone.0131248 26196693 PMC4510440

[97] Wolk K Mitsui H Witte K Gellrich S Gulati N Humme D. Deficient cutaneous antibacterial competence in cutaneous T-cell lymphomas: role of Th2-mediated biased Th17 function. Clin Cancer Res. (2014) 20:5507–16. 10.1158/1078-0432.CCR-14-0707 25212608

